# The antioxidant potential of the Mediterranean diet in patients at high cardiovascular risk: an in-depth review of the PREDIMED

**DOI:** 10.1038/s41387-018-0025-1

**Published:** 2018-03-09

**Authors:** Hayley E Billingsley, Salvatore Carbone

**Affiliations:** 1 VCU Pauley Heart Center, Virginia Commonwealth University, Richmond, VA USA; 2 Department of Experimental Medicine, Sapienza University of Rome, Rome, Italy

## Abstract

Cardiovascular disease (CVD) is the leading global cause of death. Diet is known to be important in the prevention of CVD. The PREDIMED trial tested a relatively low-fat diet versus a high-fat Mediterranean diet (MedDiet) for the primary prevention of CVD. The resulting reduction of the CV composite outcome resulted in a paradigm shift in CV nutrition. Though many dietary factors likely contributed to this effect, this review focuses on the influence of the MedDiet on endogenous antioxidant systems and the effect of dietary polyphenols. Subgroup analysis of the PREDIMED trial revealed increased endogenous antioxidant and decreased pro-oxidant activity in the MedDiet groups. Moreover, higher polyphenol intake was associated with lower incidence of the primary outcome, overall mortality, blood pressure, inflammatory biomarkers, onset of new-onset type 2 diabetes mellitus (T2DM), and obesity. This suggests that polyphenols likely contributed to the lower incidence of the primary event in the MedDiet groups. In this article, we summarize the potential benefits of polyphenols found in the MedDiet, specifically the PREDIMED cohort. We also discuss the need for further research to confirm and expand the findings of the PREDIMED in a non-Mediterranean population and to determine the exact mechanisms of action of polyphenols.

## Introduction

Cardiovascular disease (CVD) is the leading global cause of death, claiming an estimated 17.3 million lives in 2013^1^.

Oxidative stress and systemic inflammation are important contributing factors in the development and progression of CVD^2–4^. Diet, which contributed to an estimated one in two cardiometabolic deaths in the United States in 2010^5^, may play an important role in modifying oxidative stress and inflammation. However, evidence for the effects of diet on CV outcomes mainly relies heavily on observational data. Therefore, the results of the multicenter “Primary Prevention of Cardiovascular Disease with a Mediterranean Diet” trial (PREDIMED) resulted in a paradigm shift in nutrition recommendations for preventing CVD. PREDIMED tested the hypothesis that the Mediterranean diet (MedDiet) is more effective in preventing CVD than a relatively low-fat diet in a rigorous randomized-control fashion^6^.

**Fig. 1:**
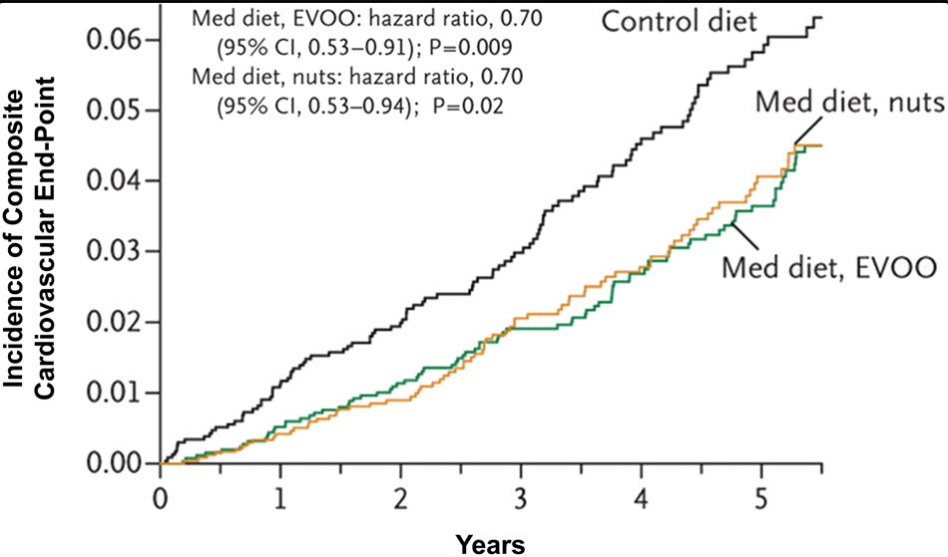
**Kaplan–Meier curve for incidence of primary outcome (composite of acute myocardial infarction, stroke, or death from cardiovascular causes) in the PREDIMED trial after a median follow-up of 4.8 years for participants.** With permission from Estruch et al.^6^

PREDIMED enrolled 7447 participants free of CVD at baseline, but at a high risk for CVD, who were randomized 1:1:1 to three groups: two caloric-unrestricted MedDiet groups receiving either ~1 liter per week of extra-virgin olive oil (EVOO) or 30 g of mixed nuts per day, and one group counseled to follow a low-fat diet^6^. The low-fat diet counseling included encouraging lean fish and poultry, low-fat dairy products, fruits, vegetables, and starches including bread, pasta, potatoes, and rice while minimizing use of oils, nuts, fatty fish and meats, commercial baked goods, and fried foods. Objective compliance to the respective Mediterranean diet intervention was tested with plasma alpha-linoleic acid (nuts group) and urine hydroxytyrosol (EVOO group) at baseline, 1, 3, and 5 years, respectively. The trial was terminated early, after interim analysis revealed a dramatic divergence for the primary end point between the groups at median follow-up of 4.8 years. Analysis revealed an impressive relative risk reduction of 30% in the primary composite outcome (acute myocardial infarction, stroke, or death from CV causes) for the two MedDiet groups compared to the low-fat control diet group (Fig. 1)^6^. The reduction of CVD was particularly evident for stroke.

It is important to note that the benefit of the Mediterranean diet in the PREDIMED trial was likely due to numerous factors, notably a high-unsaturated fat content^7, 8^, as well as the effects of other macro and micronutrients and polyphenols. It is impossible to distinguish which dietary factor led to the most benefit, most likely the summation of beneficial factors in the Mediterranean diet^9, 10^.

This review, however, will discuss the overall MedDiet tested in the PREDIMED and more specifically will focus on the ability of dietary polyphenols and vitamin antioxidants on altering oxidative stress, and most importantly, clinical outcomes.

### PREDIMED population and baseline oxidative stress level

Conducted in Spain, PREDIMED was a multicenter randomized-controlled trial that enrolled men and women 55–80 years of age with a high risk for CVD^6^. To be enrolled, participants either had type II diabetes (50%) at baseline or three or more risk factors as follows: current smoker (14%), dyslipidemia (>70%), overweight/obese (>90%), and family history of CVD (22%). When a sample of the PREDIMED cohort (*n* = 527) was compared to healthy controls (*n* = 25) before randomization, it was found that oxidative stress markers including damaged DNA base 8-oxo-dG, malondialdehyde (MDA), oxidized glutathione (GSSG), and the GSSG/glutathione (GSH) ratio were increased, revealing a more negative oxidative stress profile in the individuals with high CVD risk^11^. Increased GSSG is usually associated with early atherosclerosis in healthy adults^12^. However, in addition to oxidative stress levels, is important to analyze the endogenous antioxidant activity^13^. Endogenous antioxidant activity, measured as catalase (CAT) and glutathione peroxidase (GPx) activity, were higher in healthy subjects^11^, suggesting that in addition to lower oxidative stress levels, there was an increased antioxidant defense. Interestingly, a different sample of PREDIMED (*n* = 1069) found that increasing GPx activity was linearly associated with increasing blood glucose and oxidized Low-density Lipoprotein (LDL)^14^. The authors suggest this may be an indicator of the oxidative stress balance, likely in this case, an antioxidant response to increased presence of oxidative stress as compensatory mechanism^14^. Such findings suggest that a comprehensive assessment of oxidative stress level in association with endogenous antioxidant capacity should be recommended for a clearer picture of the overall oxidative stress/antioxidant balance.

Returning to the previous sample of 527 subjects, the presence of hypertension was the most important risk factor in determining differences in oxidative stress parameters^11^. Most of the oxidative stress measures listed above (i.e., higher GSSG, GSSG/GSH ratio, trend toward higher MDA) were increased in hypertensive subjects compared to those without hypertension. It should be noted that the activity of endogenous antioxidants GPx and superoxide dismutase (SOD) were also significantly increased in hypertensive subjects but were still at significantly lower levels than the healthy controls^11^.

This baseline data on the PREDIMED cohort demonstrates an elevated oxidative stress in individuals at high CVD risk, creating ample opportunity to explore the effect of the MedDiet.

### Effects of MedDiet on endogenous antioxidant systems

The MedDiet’s beneficial effects are usually attributed to the action of nutrients within foods consumed but the overall dietary pattern may also enhance the body’s endogenous defenses through mechanisms not fully understood^15^. A small sample (*n* = 75) of the PREDIMED cohort was analyzed for the effect of dietary intervention influence on plasmatic antioxidant and pro-oxidant activity prior to and post intervention^16^. In this study, prior to dietary intervention, EVOO- or nuts-supplemented groups presented lower plasma activity of antioxidant extracellullar SOD (EC-SOD) and catalase (CAT) activity compared to healthy controls. After intervention (median 4.8 years in entire cohort), activity of EC-SOD and CAT was enhanced in both MedDiet groups^16^. Additionally, pro-oxidant xanthine oxidase (XOX) had decreased activity in both MedDiet groups. When further analyzed for degree of adherence as measured by questionnaire, plasma antioxidant activity of EC-SOD and CAT increased with greater adherence to the MedDiet, while pro-oxidant XOX activity decreased with greater MedDiet adherence^16^. However, no differences were seen in plasma markers of oxidative damage and no differences were seen in functionality of serum myeloperoxidase or whole blood and blood cell EC-SOD and CAT^16^. When total antioxidant capacity (TAC) of serum was measured in another subset of PREDIMED participants (*n* = 187), the subjects randomized to EVOO-supplemented MedDiet and nuts-supplemented MedDiet had greater TAC than the subjects randomized to the relatively low-fat control diet^17^. Within EVOO- and nuts-supplemented MedDiet groups, EVOO-supplemented MedDiet induced an even further increase in TAC compared to the nuts-supplemented MedDiet^17^. In an additional analysis of this substudy, the TAC was also associated with weight loss in the EVOO intervention group, suggesting that greater TAC may be involved in body weight regulation. However, a major limitation of this additional analysis was that total caloric intake was not included in the multivariate^17^, therefore we cannot determine whether such association was independent of total energy intake. From this subanalysis, however, it is not possible to determine whether TAC directly affected body weight, or viceversa, suggesting the need for further study.

### Importance of dietary antioxidants and polyphenols

Dietary vitamin antioxidants and polyphenols have been explored extensively as an exogenous mechanism of defense against oxidative stress, systemic inflammation, and finally, the development of non-communicable disease^18–20^. Increased dietary antioxidant content (i.e., vitamins and polyphenols) has been associated with reduced incident of heart failure^21^, stroke^22^, coronary artery disease^23^, and cancer^24^. It should be emphasized that this does not include antioxidant supplementation, which does not decrease mortality^25^.

The MedDiet is rich in both vitamins and polyphenols, which are mainly, but not exclusively contained in fruits, vegetables, whole grains, nuts, EVOO, and red wine^26^. The traditional vitamin antioxidants are usually beta-carotene (vitamin A precursor), vitamins C, vitamin E, and often the mineral selenium^27^. In contrast, polyphenols encompass a wide range of secondary plant metabolites characterized by a large number of phenol groups^28^. Polyphenols are produced in response to a threat (e.g., radiation, pathogens)^29^ and it has been hypothesized that their protective ability may also have importance in the human body^30^. Multiple hydroxyl groups make polyphenols strong antioxidants but they also carry anti-inflammatory properties^31^. In fact, they can interfere with a number of proinflammatory pathways such as the Nod-like receptor pyrin domain-containing protein (NLRP)3 inflammasome, nuclear factor kappa B (NFkB), and mitogen-activated protein kinase (MAPK)^28, 31^. Particularly, in vitro studies suggested that the inhibition of these transcription factors or macromolecular complexes induced by polyphenols may reduce the synthesis and release of proinflammatory cytokines such as interleukin(IL)-1β, IL-6, IL-8, and tumor necrosis factor (TNF-a), which have in turn, been associated with adverse CV events^28, 32^. However, their powerful in vitro anti-inflammatory effects may not necessarily translate to the same beneficial effects in vivo, as polyphenols bioavailability varies quite considerably^33^.

### Effect of dietary antioxidants

An analysis of the PREDIMED focused on total dietary antioxidant capacity—including all compounds with antioxidant potential (vitamin antioxidants and polyphenols)^34^. The quantity of both polyphenols and vitamin antioxidants was determined by dietary intake information collected by validated 137-question food frequency questionnaire (FFQ) for all participants at baseline and then once annually during follow-up^6^. Total dietary antioxidant capacity was then determined by converting the FFQ to the 24-h dietary recalls and then using a database with antioxidant potential of each food determined by a specific assay^34^. In this analysis of the entire PREDIMED cohort, those who where in the highest quintile of total antioxidants consumption presented a non-significant 15 and 21% relative risk reduction in overall and CV mortality for PREDIMED, respectively. There was no correlation found between antioxidant consumption and cancer-related mortality^34^.

### Effect of dietary polyphenols

While the role of vitamin antioxidants in the PREDIMED was, to date, only discussed in one study^34^, the effects of polyphenols were far more extensively studied. At baseline, for the 7200 PREDIMED subjects who completed a FFQ, the main source of polyphenols was fruit, followed by non-alcoholic beverages (i.e., coffee, orange juice, tea), and vegetables^35^. As for individual sources, coffee (18%) was the main food source of total dietary polyphenols, followed by oranges (16%), apples (12%), olives and olive oil (11%), and lastly, red wine (6%). However, this substudy did not assess potential changes of polyphenols sources over time, which may have occurred when participants were randomized into the dietary intervention groups^6^. Of note, PREDIMED was conducted in a Mediterranean country (Spain), which likely influenced baseline dietary pattern and therefore sources of polyphenols.

**Fig. 2:**
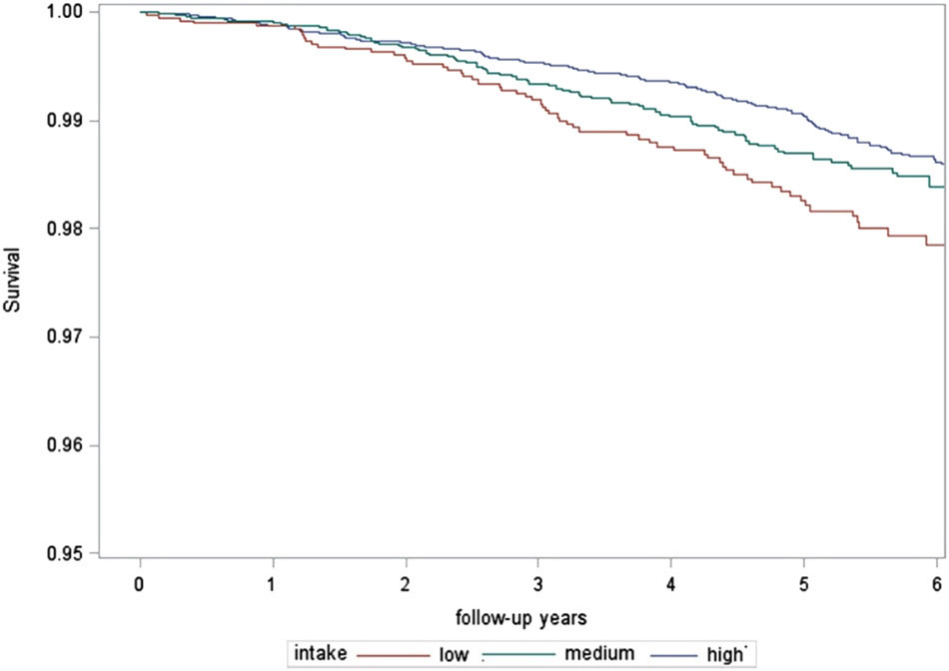
**Nelson Aalen survival function of the PREDIMED cohort divided into tertiles of polyphenol consumption.** With permission from Tressera-Rimbau et al.^37^

In another analysis of PREDIMED participants (*n* = 7172), which only included those with a baseline FFQ and plausible energy intake, a multivariate analysis adjusting for age, BMI, smoking status, physicial activity, and energy intake, as well as selected nutrient intake, medications and CV risks, found that individuals in the highest quintile vs. the lowest quintile of polyphenols intake measured by FFQ had a significant 46% relative risk reduction in the primary composite CV outcome^36^. However, when the three randomized groups were analyzed individually, this association was only apparent in the control group assigned to the relatively low-fat diet^36^. The authors postulated that this was possibly influenced by the relatively small event rate in both MedDiet intervention groups^36^. Interestingly, the subjects in the highest quintile of polyphenols presented a relative mortality risk reduction of 37% compared to those in the lowest quintiles of polyphenols intake^37^. However, the authors noted this was not a strong linear association and the association lessened after the lowest quintile of polyphenol intake was removed from the analysis. Figure 2 demonstrates the separation in crude mortality rates of the partcipants divided into tertiles by low, medium, and high consumption of polyphenols^37^. When looking at the individual polyphenols, the polyphenols associated with the greatest all-cause mortality reduction were stilbenes and lignans^37^. Such results were confirmed also in relation to the primary outcome, indeed the top quintile of consumption of the polyphenol lignan was associated with a 49% relative risk reduction of the composite CV primary outcome of the study^36^. Although the mechanisms underlying such improvements are still largely unknown, lignans exert a number of potential beneficial effects such as anti-inflammatory, antioxidant, and anti-tumor properties^38^. The greatest source of lignans in the PREDIMED cohort was EVOO^34^ and another PREDIMED analysis demonstrated a potential ability of the polyphenols present in EVOO to downregulate the expression of pro-atherosclerosis genes and related inflammatory and lipid oxidative markers^39^.

Of note, polyphenol content can differ dramatically between different olive oil products. EVOO has the highest content of polyphenols with four main groups of phenol compounds: simple phenols (hydroxytyrosol), flavonoids, secoiridoids, and lignans^40^. Hydroxytyrosol is particularly important also due to its urinary excretion, which allows to measure compliance to EVOO consumption^41^. Moreover, hydroxytyrosol has the highest antioxidant potential of the phenol compounds contained in olive oil, at least in vitro^39^.

The influence of polyphenols on inflammatory markers and CV risk factors was also investigated. Total polyphenol excretion (TPE) in the urine is a validated method of dietary polyphenol quantification^42^. One substudy of randomly selected PREDIMED subjects (*n* = 1139) demonstrated that individuals in the two MedDiet cohorts (EVOO and nuts) significantly increased their TPE after 1 year of intervention and decreased systemic inflammatory biomarkers from baseline including circulating vascular cell adhesion molecule 1, (VCAM-1), soluble inter cellular adhesion molecule 1 (ICAM-1), IL-6, TNF-α, and monocyte chemotactic protein 1 (MCP-1)^43^. Conversely, these molecules with potential detrimental effects increased in the control group, although the difference did not reach statistical significance^43^. When all patients were separated into tertiles based on changes in TPE, a difference was demonstrated between the highest and lowest tertiles of increase in TPE and the change in levels of the inflammatory biomarkers listed above (i.e., VCAM-1, ICAM-1, IL-6, TNF- α, MCP-1)^43^.

The substudies and analyses contained in this section present the possibility that polyphenol intake is linked to systemic inflammation and overall mortality. However, it must be taken into consideration that the polyphenol analyses^36, 37^ were extrapolated from the main study^6^, which was not initially designed to look at the effects of polyphenols. Moreover, although the investigators performed several statistical adjustments to the multivariate analysis to minimize the potential of confounders, it is possible that other confounding factors existed and for which the statistical analysis could not fully account for. Additionally, such studies cannot prove causation, therefore future randomized-controlled trial modulating polyphenols intake are warranted to confirm these effects.

### TPE and blood pressure

A cross-section substudy of PREDIMED performed at baseline (*n* = 589), demonstrated that patients in the highest quintile of urinary TPE had 36% lower risk of having hypertension compared to the lowest TPE quintile, even after multivariate analysis, which included gender, age, weight, smoking, physical activity, education, medication use, sodium/potassium intake, and glomular filtration rate^44^. The interaction between blood pressure, nitric oxide (NO), and polyphenols measured by TPE was also assessed in another sample (*n* = 200) after 1 year of dietary interventions^45^. In this particular substudy, intervention groups displayed a significant lowering of systolic blood pressure −5.79 mmHg and −7.26 mmHg with MedDiet with EVOO and nuts, respectively, after 1 year of follow-up^45^. In the entire PREDIMED cohort, this effect was less preminent, and no clinically significant diastolic blood pressure-lowering was observed in the MedDiet groups versus the control after 4 years of follow-up^46^. Higher quintiles of TPE were also associated with increased NO levels and the authors attributed the blood pressure-lowering effects of the MedDiet to the action of the polyphenols on increasing NO, therefore causing vasodilation, as previously shown in vitro^45^.

The studies presented above suggest that polyphenol intake may have a blood pressure-lowering effect^44, 45^. However, it should be noted that both studies^44, 45^ are very small samples of the PREDIMED cohort^46^.

### TPE, incident type 2 diabetes mellitus, and obesity

Incidence of type 2 diabetes mellitus (T2DM) was a pre-specified outcome of the PREDIMED trial^47^. A subgroup analysis of 3541 participants showed a 52% relative risk reduction of T2DM after a median follow-up of 4.0 years in the combined MedDiet groups, even in absence of body weight and physical activity changes over the duration of the study^48^. A significant reduction of new-onset T2DM was also found in those with the highest (versus the lowest) baseline intake of polyphenols after 5.5 years of follow-up in 3430 patients free from T2DM at baseline^49^. Additionally, in a cross sectional substudy (*n* = 573), a significant inverse correlation was found between new-onset obesity and individuals in the highest versus lowest quintile of polyphenol intake measured by urinary TPE^50^. Protection against weight gain is one plausible mechanism by which higher dietary polyphenols may help prevent new-onset T2DM, but a recent review also suggested several mechanisms for the association between polyphenols and reduction in T2DM risk including improved insulin sensitivity, reduction of oxidative stress, and systemic low-grade inflammation^51^.

It is important to note in the two studies mentioned above assessed polyphenols differently—one by dietary intake^49^ and the other by TPE^50^. Additionally, all three studies mentioned contain 50% or less of the total PREDIMED cohort^48–50^.

## Discussion

Individuals with high CV risk who consume high amounts of polyphenols may present a lower risk of developing CV events and mortality by potential modulation of risk factors such as systemic inflammation^43^, obesity^50^, blood pressure^44, 45^, and T2DM^49^.

Only one substudy of the PREDIMED analyzed the effects of vitamin antioxidants on major CV events^34^, so their individual contribution to the different end points of the study cannot be determined.

It is acknowledged that the evidence regarding polyphenols presented in the above paper was not a pre-specified outcome of the orginal PREDIMED trial. The studies presented are substudies and analyses of various sizes and methodologies, and the results should be interpreted with caution and used for hypothsis-generating purposes. Morevoer, while multivariate adjustment is performed in most of statistical analyses, it is impossible to fully adjust for every possible confounding factor. Additionally, causation cannot be proven by such studies and appropriately powered randomized-controlled dietary trials of polyphenols modulation are required to expand these observations.

**Fig. 3:**
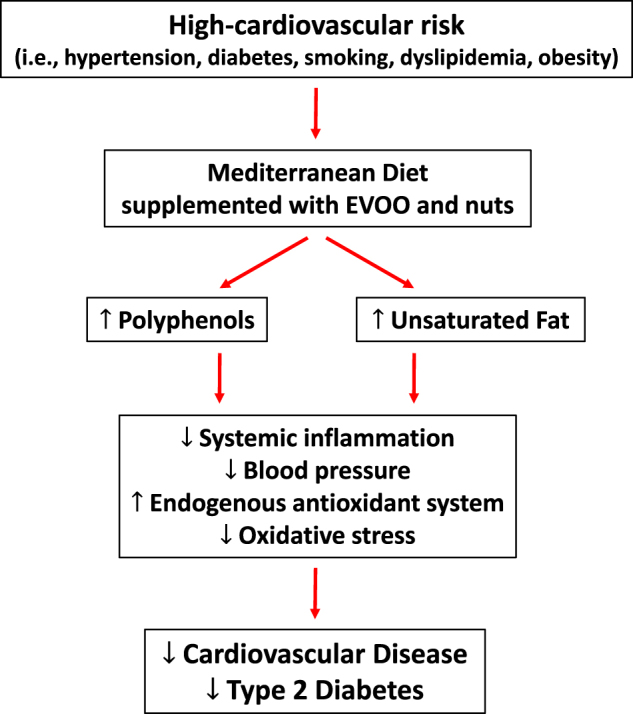
**The figure shows the relationship between Mediterranean diet and decreased incidence of cardiac events and type 2 diabetes in the PREDIMED cohort and the suggested underlying mechanisms of action of the Mediterranean diet**

Polyphenols often are consumed in the form of fruits, vegetables, and other plant-derived food products^52^ that have nutrients such as dietary fiber with established health benefits^53^, but also EVOO^54^ and nuts^55^. Within the limitation of the studies analyzed herein (i.e., limited sample size, subgroup analysis), the evidence would suggest that polyphenols are a powerful piece of the puzzle that makes the MedDiet a powerful prevention tool in the hands of the provider (Fig. 3). In addition to polyphenols, however, the MedDiet is also very rich in unsaturated fatty acids (UFA). It has been increasingly recognized that a diet high in UFA is extremely important for CV health^56^. However, food rich in UFA are often also rich in polyphenols (i.e., EVOO), making it very difficult to differentiate the beneficial individual effects of one component or the other. The difficultly of disentangling the beneficial effects of the dietary intervention versus the more comprehensive Mediterranean dietary pattern has been fully acknowledged by the PREDIMED investigators^57^. It is fully possible that a combination of the polyphenols and UFA is required for a more powerful beneficial effect (Fig. 3).

Taken together, these data presented in this review can also serve as evidence toward what the ideal MedDiet for the prevention of chronic disease may look like as the phrase “Mediterranean diet” describes a pattern of eating that may vary regionally among the Mediterranean countries^58^. Additionally, this could inform the updates of dietary guidelines to help consumers and health professionals better understand how to instruct and comply with the MedDiet as dietary patterns are being increasingly emphasized over individual foods and nutrients in the United States Dietary guidelines^59^.

A notable limitation of the PREDIMED is the minimal guidance the low-fat diet participants received in the first 3 years of the trial. Though both groups received baseline dietary counseling, the MedDiet groups received follow-up in the form of personalized, quarterly counseling but the low-fat diet participants only received an annual informational pamphlet. This was admended due to the concern for bias and low-fat diet participants began receiving personalized counseling as frequently as the MedDiet groups in 2006. Importantly, after analysis, no difference in benefit was demonstrated with respect to enrollment period between the MedDiet and the low-fat groups suggesting the initial lack of counseling intensity did not influence results. Another major limitation of the PREDIMED is that the study was performed only in Spain, in a population with a diet that is partly already reflective of the MedDiet. There is therefore an urgent need to explore EVOO and nut supplementation in a non-Mediterranean population^60^. Recently, the National Heart, Lung, and Blood Institute created a working group in conjunction with the National Cancer Institute and Office of Disease Prevention to address this interest in the context of a MedDiet intervention in the American population^61, 62^. Reflecting on the compelling evidence presented above for the beneficial effects of a polyphenol-rich Mediterranean diet, this is much needed for the future of preventative CV care in America.

The accumulation of evidence in support of the Mediterranean diet for optimal health continues to grow^63^. Overall, mortality and cardiometabolic mortality/events were focused on in PREDIMED, but evidence also suggest potential benefit in cognitive health^64, 65^, cancer^66^, and even incidence of fractures^67^. It is now clear that in the PREDIMED cohort the MedDiet supplemented with nuts or EVOO reduces cardiac events. The evidence presented above shows that polyphenols likely contribute to this effect through lowering inflammatory biomarkers, blood pressure and reduction of new-onset T2DM, and obesity. Although polyphenols play an important role in affecting CVD as described in this review, the high-unsaturated fat content of the Mediterranean diet also plays a crucial role in preventing CVD. In fact, a recent presidential advisory from the American Heart Association^68^ recommended to increase the consumption of UFA, and substituting saturated fatty acids for UFA to prevent CVD, without specific recommendation on the amount of calories deriving from total fat or UFA.

To date, however, we do not have enough evidence to determine whether the high polyphenol content or the high UFA content is the major driver of CVD prevention, perhaps a combination of the two may exert the greatest benefits.

## Conclusion

In conclusion, the high polyphenols content of the MedDiet and the increase in the overall antioxidant endogenous system seen in patients assigned to supplementation of EVOO and nuts, may explain, at least in part, the beneficial effects reported in the PREDIMED. However, the studies presented herein have limitations, and future study is needed to determine whether polyphenols or UFA play a major role in preventing CVD and metabolic disease, and importantly, whether the overwhelming beneficial effects of the MedDiet can be replicated in a non-Mediterranean countries.
